# 

**Published:** 1868-01

**Authors:** 


### CONTENTS.

### Number XI.

### ORIGINAL COMMUNICATIONS.

PAGE.

ARTICLE I.—Carbuncle: Its Pathology and Treatment. By John M. Johnson, M. D., of Atlanta ... 461

ARTICLE II.—A Fatal Case of Unnatural Labor. By W. C. Bellamy, of Columbus, Georgia ... 464

ARTICLE III.—Gonorrhœa; Its Treatment. By John M. Johnson, M. D., of of Atlanta, Ga ... 469

ARTICLE IV.—Galium. By D. L. Phares, M. D., of Newtonia, Miss ... 470

European Correspondence ... 474

### Number XII.

### ORIGINAL COMMUNICATIONS.

PAGE.

ARTICLE I.—Rubiaceæ: By D. L. Phares, M. D., of Newtonia, Miss ... 495

ARTICLE II.—Bromine and its Preparations in Diseases. By J. J. Knott, M. D., of Atlanta, Ga ... 503

Bibliographical ... 505

Editorial and Miscellaneous ... 514

To the Members of the Medical Association of the State of Georgia ... 520

Atlanta Board of Health ... 520

Special Notice ... 521

Receipts for the Journal ... 521

